# Effect of aerobic exercise and diet on liver fat in pre-diabetic patients with non-alcoholic-fatty-liver-disease: A randomized controlled trial

**DOI:** 10.1038/s41598-017-16159-x

**Published:** 2017-11-21

**Authors:** Sulin Cheng, Jun Ge, Can Zhao, Shenglong Le, Yifan Yang, Dandan Ke, Na Wu, Xiao Tan, Xiaobo Zhang, Xiaming Du, Jianqin Sun, Renwei Wang, Yongyong Shi, Ronald J. H. Borra, Riitta Parkkola, Petri Wiklund, Dajiang Lu

**Affiliations:** 1 Exercise, Health and Technology Centre, Department of Physical Education, Shanghai Jiao Tong University, 200240 Shanghai, China; 2 Shanghai Yangpu district Shidong Hospital, 200090 Shanghai, China; 3 School of Kinesiology, Shanghai University of Sport, 200438 Shanghai, China; 4 Faculty of Sport and Health Sciences, University of Jyväskylä, 40014 Jyväskylä, Finland; 5 The Key Laboratory of Systems Biomedicine, Ministry of Education, Shanghai Center for Systems Biomedicne, Shanghai Jiao Tong University, 200240 Shanghai, China; 6 Clinical Nutrition Centre, Fudan University Huadong Hospital, 200040 Shanghai, China; 7 Bio-X, Shanghai Jiao Tong University, 200030 Shanghai, China; 8 Department of Diagnostic Radiology, University of Turku and Turku University Hospital, Turku, Finland; 9 Department of Nuclear Medicine and Molecular Imaging, University of Groningen, University Medical Center Groningen, Groningen, The Netherlands; 10 Department of Epidemiology and Biostatistics, Centre for Environment and Health, School of Public Health, Imperial College London, London, UK; 11 Center for Life-Course Health Research, Faculty of Medicine, University of Oulu, Oulu, Finland

## Abstract

The study aimed to assess whether aerobic exercise (AEx) training and a fibre-enriched diet can reduce hepatic fat content (HFC) and increase glycaemic control in pre-diabetic patients with non-alcoholic fatty liver disease (NAFLD). Six-hundred-and-three patients from seven clinics in Yangpu district, Shanghai, China were recruited. Of them 115 individuals aged 50–65-year fulfilled the inclusion criteria (NAFLD with impaired fasting glucose or impaired glucose tolerance) and were randomly assigned into exercise (AEx n = 29), diet (Diet n = 28), exercise plus diet (AED n = 29), or no-intervention (NI n = 29) groups. Progressive supervised AEx training (60–75% VO2max intensity) was given 2-3 times/week in 30–60 min/sessions, and the diet intervention was provided as lunch with 38% carbohydrate and diet fibre of 12 g/day for 8.6-month. HFC was assessed by ^1^H MRS. We found that HFC was significantly reduced in the AEx (−24.4%), diet (−23.2%), and AED (−47.9%) groups by contrast to the 20.9% increase in the NI group (p = 0.001 for all) after intervention. However, only AED group significantly decreased HbA_1c_ (−4.4%, p = 0.01) compared with the NI group (−0.6%). Aerobic exercise training combined with fibre-enriched diet can reduce HFC more effectively than either exercise or increased fibre-intake alone in pre-diabetic patients with NAFLD.

## Introduction

Non-alcoholic fatty liver disease (NAFLD) is the most common cause of chronic liver disease in the developing world, and occurs in 17–30% of the population in Western countries and 2–4% worldwide^1^. In China the prevalence of NAFLD is 10–30% and up to 75% among obese and type 2 diabetes (T2D)^2^. At the same time, a recent large national survey estimated that 10% of Chinese adults may have diabetes and 50% may have prediabetes^3^. NAFLD and prediabetes often co-exist and may synergistically increase the risk of both T2D and cardiovascular diseases^4^. Finding effective ways to improve glycaemic control and hepatic steatosis are therefore urgently needed.

Previous studies have shown that calorie restriction^5^ and exercise are effective ways to improve hepatic steatosis^6^ and insulin sensitivity^7^, and are therefore recommended as a first-line interventions for prevention and treatment of NAFLD and pre-diabetes^8^. Furthermore, several randomized controlled trials have shown that increased fibre intake improves glycaemic control in patients with T2D^9^, suggesting that it could be used as an additional tool to improve long-term glucose control. However, to date, no randomized control study has evaluated the effect of combined aerobic exercise and fibre-enriched diet on reduction of HFC and glycaemic control in people with both pre-diabetes and NAFLD.

Recent systematic reviews of randomized controlled trials indicate that exercise interventions may improve glycaemic control in subjects with impaired glucose tolerance and T2D^10,11^. Systematic reviews have also shown that exercise can reduce hepatic steatosis in patients with NAFLD, independent of weight loss and dietary intake^12,13^. In addition, a recent systematic review and meta-analysis of the effect of fibre intake on glycaemic control in patients with T2D showed that increased fibre intake may improve glycaemic control, and thus could be used as an adjunctive tool in the treatment of patients with dysglycemia^9^.

In summary, although exercise or dietary intervention alone showed effective benefit on glycaemic control and NAFLD reduction, no study so far has assessed the combined effect of both exercise and high fibre intake on glycaemic control and hepatic steatosis in people with pre-diabetes and NAFLD. Therefore, we aimed to test our hypothesis that exercise training combined with increased fibre intake reduces hepatic steatosis and improves glycaemic control in pre-diabetic patients with NAFLD. The study has been registered in the International Standard Randomized Controlled Trial Number Register (ISRCTN 42622771, Date applied 10/04/2013, Date assigned, 23/04/2013, Last edited 02/02/2017).

## Results

### Participants and baseline characteristics

**Figure 1:**
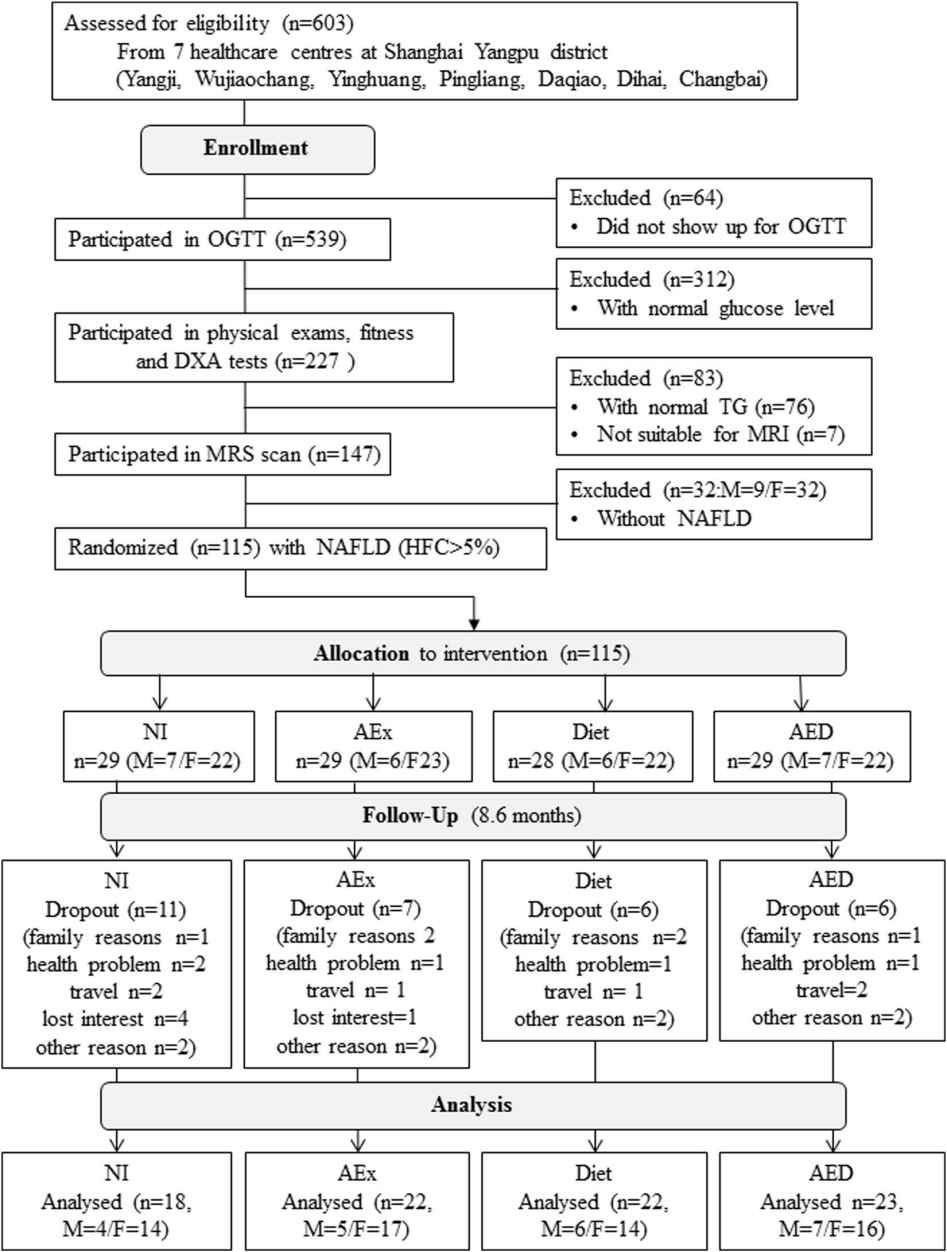
Study profile. HFC = Hepatic fat content; NAFLD = non-alcoholic fatty liver disease; AEx = aerobic exercise; AED = exercise + diet; NI = no intervention, OGTT = oral glucose tolerance test; TG = glucose; MRS = proton magnetic resonance spectroscopy; DXA = dual X-ray densitometry; M = male; F = female.

An overview of the study is given in Fig. 1. The study was performed between January 6 and December 25, 2013. We screened 603 individuals, of whom 115 were eligible and randomly assigned to the AEx (n = 29), Diet (n = 28), AED group (n = 29), or NI group (n = 29). Of the 115 subjects, 85 subjects (74%) completed the intervention (n = 18 in NI group, n = 22 in AEx, n = 22 in Diet, n = 23 in AED, respectively, see Fig. 1). The overall compliance was 62.1% for the NI group, 75.9% for the AEx group, 78.6% for the Diet group and 79.3% for the AED group, respectively. The main reasons for drop-out were the following: family reasons (n = 6); diseases not related to the present trial (n = 5); loss of interest (n = 5); travel (n = 6) and other personal reasons (n = 6). The high drop-out in the NI group was mainly due to loss of interest (disappointment at not being in the intervention groups).

The baseline characteristics were similar in all groups (Table 1). In addition, no significant group differences were found in the primary outcomes (HFC, HbA1c and insulin sensitivity), anthropometrics, body composition, FM and FM distribution, level of physical activity, dietary intake, or serum biomarkers. The proportion of subjects with different variants of the patatin-like phospholipase domain-containing protein 3 **(**PNPLA3, GG, GC and CC) were also similar between groups. The proportion of subjects with impaired fasting glucose (IFG) and impaired glucose tolerance (IGT) was also similar between the groups.

**Table 1:** Baseline physical characteristics, background information, body composition and adiposity in different intervention groups (Mean and SD values in bracket).

Variables	NI (n = 29)	AEx (n = 29)	Diet (n = 28)	AED (n = 29)
Sex (M/F, n)	7/22	6/23	6/22	7/22
Age (year)	60 (3.4)	59(4.4)	60 (4.1)	60 (3.5)
Height (cm)	160 (8.3)	163 (9.8)	161 (6.4)	163 (7.2)
Weight (kg)	69.5 (11.9)	72.3 (10.6)	68.7 (9.2)	70.3 (8.8)
BMI (%)	27.1 (2.9)	27.3 (3.6)	26.6 (2.7)	26.4 (2.9)
FM_WB_ (kg)	26.1 (6.2)	27.4 (6.0)	25.5 (4.3)	25.8 (4.6)
LM_WB_ (kg)	42.3 (7.5)	44.7 (8.6)	43.2 (7.6)	44.5 (7.2)
Fat_WB_ (%)	38.0 (4.3)	38.2 (5.9)	37.3 (5.0)	36.7 (5.0)
FM_Android_ (kg)	2.7 (0.7)	2.8 (0.8)	2.6 (0.5)	2.5 (0.6)
FM_Gynoid_ (kg)	3.5 (1.2)	3.7 (1.1)	3.4 (0.7)	3.4 (0.9)
VAT (kg)	1.8 (0.9)	1.7 (0.5)	1.6 (0.8)	1.7 (0.9)
SAT (kg)	2.3 (0.9)	2.6 (1.1)	2.2 (0.7)	2.1 (0.8)
HFC (%)	16.0 (9.8)	17.1 (13.1)	16.6 (11.4)	17.2 (12.9)
HbA_1C_ (%)	6.2 (0.5)	6.2 (0.4)	6.0 (0.4)	6.0 (0.5)
Glucose fasting (mmol/L)	5.7 (0.6)	5.7 (0.6)	5.3 (0.8)	5.5 (0.6)
Glucose 2 h (mmol/L)	8.1 (1.4)	7.9 (1.3)	7.9 (1.9)	8.3 (1.4)
Insulin fasting (mU/L)	17.2 (8.1)	18.8 (11.2)	14.1 (7.1)	15.8 (8.3)
Insulin 2 h (mU/L)	131 (61)	125 (84)	115 (79)	128 (82)
Triglyceride (mmol/L)	2.3 (1.9)	2.0 (1.3)	2.0 (1. 5)	2.2 (1.1)
FFA (mmol/L)	0.60 (0.3)	0.56 (0.2)	0.60 (0.2)	0.55 (0.3)
ALT (U/L)	22.0 (9.1)	21.1 (10.1)	19.8 (10.7)	22.4 (10.6)
GGT (U/L)	33.3 (17.4)	26.2 (8.9)	24.0 (14.2)	25.9 (11.1)
AST (U/L)	23.3 (8.1)	28.1 (12.8)	22.5 (9.6)	26.8 (9.3)
PNPLA3 (GG/GC/CC, %)	39/29/32	44/41/15	26/41/33	26/33/41
IFG/IGT/IFG + IGT (%)	24/35/41	31/38/31	21/57/22	21/48/31
N/PDB/DB* (%)	14/66/20	10/73/17	14/75/11	10/73/17
VO2max (ml/kg/min)	16.2 (5.4)	15.1 (5.4)	17.8 (4.3)	16.0 (5.1)
Exercise (time/wk)	2.7 (1.8)	2.3 (1.4)	3.2 (1.8)	2.8 (1.8)
Exercise (hour/wk)	2.0 (1.5)	1.9 (1.3)	3.0 (2.1)	2.6 (2.0)
IE (Kcal/d)	1735 (490)	1767 (542)	1768 (547)	1787 (620)
Fibre (g/d)	8.7 (5.1)	8.8 (4.6)	12.2 (5.7)	10.7 (5.3)
Protein (E%)	22.0 (4.3)	19.3 (4.2)	20.3 (3.6)	20.5 (5.9)
Carbohydrate (E%)	47.9 (11.5)	50.9 (12.6)	51.3 (10.3)	51.9 (9.6)
Fat (E%)	32.2 (9.9)	30.7 (11.0)	29.1 (8.4)	28.4 (8.4)
Alcohol use (N/Y, %)	79/21	72/28	82/18	79/21
Smoking (N/Y, %)	86/14	90/10	93/7	97/3

ANOVA followed by Sidak between the groups.

SD = Standard deviation, NI = No intervention group, AEx = Exercise group, AED = AEx + diet group, BMI = Body mass index, FM = Fat mass, FM_WB_ = Fat mass of the whole body, LM = Lean mass, HFC = Hepatic fat content, VAT = Visceral adipose tissue, SAT = Subcutaneous adipose tissue, PNPLA3 = Patatin-like phospholipase domain containing 3, VO2max = maximum oxygen uptake, EI = Energy Intake, HbA_1c_ = HemoglobinA1c, FFA = Free fatty acid, ALT = Alanine aminotransferase, GGT = γ-Glutamyltransferase, AST = Aspartate aminotransferase, IFG = impaired fasting glucose, IGT = impaired glucose tolerance, *defined by HBA_1c_ for N = normal (HbA_1c_ < 5.7%, PBD = prediabetes HbA_1c_ 5.7% −6.4%, DB = diabetes HbA_1c_ > 6.4%).

### Primary outcomes of efficacy analysis

**Figure 2:**
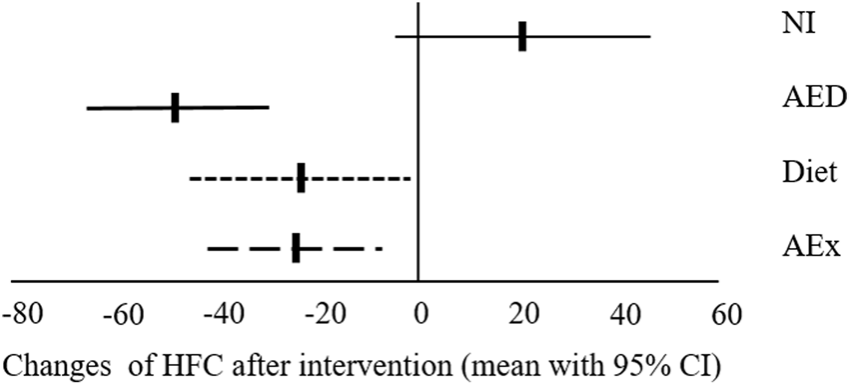
Change of hepatic fat content (HFC) after intervention. AEx = aerobic exercise; AED = exercise + diet; NI = no intervention; CI = confident interval.

The mean percent changes in HFC from baseline to 8.6 months in different groups are shown in Fig. 2. HFC decreased in all intervention groups compared with the NI group. Mean change in HFC was −24.4% (95% CI −41.7 to 7.1) in the AEx group, −23.2% (−45.1 to −1.3) in the diet group, and −47.9% (−65.8 to −30.0) in the AED group, and 20.9% (−4.4 to 46.2) in the NI group (p = 0.006, p = 0.002, and p < 0.0001, respectively, Fig. 2). After controlling for change of body weight, duration of the intervention and baseline HFC, the significant differences remained with effect size of η = 0.159 (p = 0.006, AEx vs. NI p = 0.02; Diet vs. NI p = 0.017 and AED vs. NI p = 0.001, respectively). In total, HFC decreased in 91% of the subjects in the AED group, and the corresponding figures were 68% in the AEx group, 86% in the Diet group (Table 2). In contrast, 72% in the NI group increased their HFC during the intervention period. Importantly, HFC decreased to normal levels (<5.6%) in ten (44%) of 23 participants in the exercise plus diet group, three (14%) of 22 participants in the exercise group, and nine (41%) of 22 participants in the diet group (Table 2).

**Table 2:** Remission and progression of NAFLD and prediabetes after intervention^a^.

		NI	AEx	Diet	AED	χ^2^
		(n = 18)	(n = 22)	(n = 22)	(n = 23)	p-value
NAFLD*	(n, 1/2/3)	2/3/13	3/12/7	9/10/3	10/11/2	0.001
	(%,1/2/3)	11/17/72	14/54/32	41/45/14	43/48/9	
HbA_1c_^†^	(n, 1/2/3)	1/12/5	2/18/2	5/14/3	3/17/3	0.454
	(%, 1/2/3)	6/67/28	9/82/9	23/64/14	13/74/13	
IFG^†^	(n, 1/2/3)	7/9/2	14/8/0	10/12/0	10/12/1	0.322
	(%, 1/2/3)	39/50/11	64/36/0	46/54/0	44/52/4	
IGT^†^	(n, 1/2/3)	7/8/3	13/5/4	11/9/2	12/8/3	0.786
	(%, 1/2/3)	39/44/17	59/23/18	50/41/9	52/35/13	

*1 = back to normal (HFC < 5.6%); 2 = decreased; 3 = increased.

^†^1 = back to normal (HBA1c < 5.7%, fasting glucose < 5.6 mmol/l, 2h-glucose < 7.8 mmol/l); 2 = maintained prediabetes (HBA1c 5.7–6.4%, fasting glucose ≥5.6–6.9 mmol/l, 2h-glucose ≥7.8–11.0 mmol/l); 3 = become diabetes (HBA1c ≥ 6.5%, fasting glucose ≥7.0 mmol/l, 2h-glucose ≥11.0 mmol/l).

^a^All the participants who have been in the final assessments.

### Secondary outcomes of efficacy analysis

**Figure 3:**
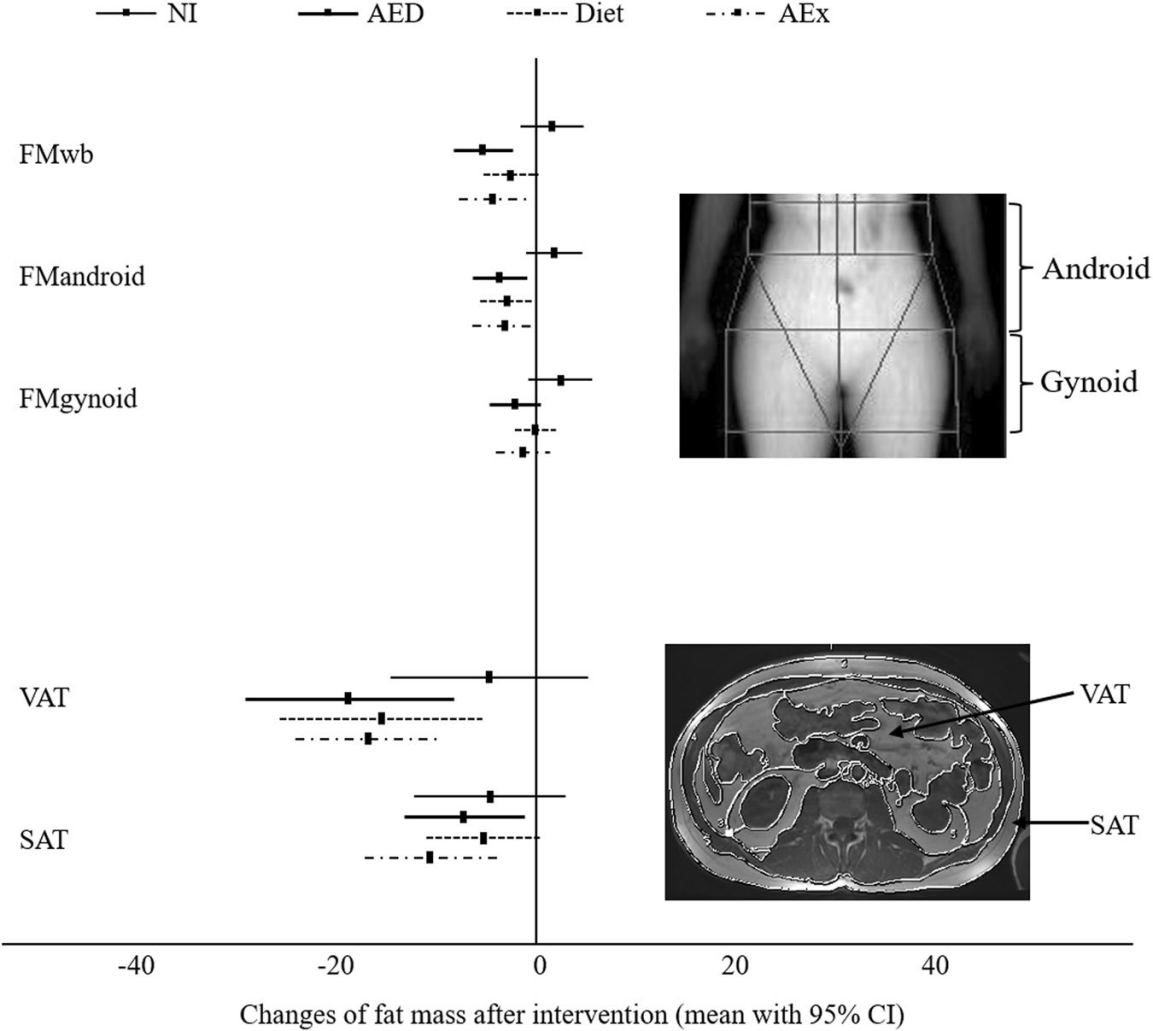
Change of central body fat mass after intervention. AEx = aerobic exercise; AED = exercise + diet; NI = no intervention; FMwb = fat mass of the whole body; FM_android_ = fat mass of the android region; FM_gynoid_ = fat mass of the gynoid region; VAT = visceral adipose tissue; SAT = abdominal subcutaneous adipose tissue. The scan pictures were taken from one of the researchers as quality control during the study.

The percentage changes of the FM in different compartments of the body are shown in Fig. 3. Compared to the NI group, the AED and AEx groups decreased significantly their FM of the whole body (FMwb, p = 0.001 and p = 0.002) and in the android region (p < 0.001 and p = 0.006). The diet group also lost more their FM in the android region (p = 0.009) and tended towards lower FM in the whole body (p = 0.051). Both AED and AEx groups also had reduced FM in gynoid region compared with the NI group (p = 0.025 and p = 0.028). Visceral (VAT) and subcutaneous (SAT) FM decreased with time in all intervention groups (VAT: AEx p < 0.001; Diet p = 0.007; AED p = 0.019, SAT: AEx p = 0.005; Diet p = 0.075; AED p = 0.063), but not in the NI group. However, no significant group-by-time differences were found.

**Figure 4:**
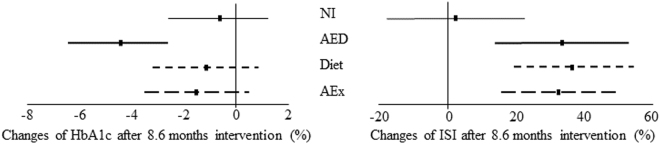
Change of HbA_1c_ and insulin sensitivity after intervention. AEx = aerobic exercise; AED = exercise + diet; NI = no intervention; CI = confident interval; HBA_1c_ = glycated haemoglobin; ISI = insulin sensitivity index.

Furthermore, we found that glycated haemoglobin (HbA_1c_) decreased significantly in the AED group compared to the NI group (p = 0.01), Diet (p = 0.027) and AEx (p = 0.049) groups **(**Fig. 4a **)**. The change of HbA_1c_ was −1.5% with 95% CI of −3.5 to 0.5 in the AEx group, −1.1% with 95% CI of −3.2 to 0.95 in the diet group, −4.4% with 95% CI of −6.4 to −2.4% in the AED group and −0.65% with 95% CI of −2.6 to 1.3% in the NI group, whereas insulin sensitivity index (ISI) increased in all intervention groups compared to the NI group (AEx: 33%, p = 0.023, Diet: 37%, p = 0.012 and AED: 34%, p = 0.029, respectively, Fig. 4b). However, after controlling for the change of body weight, duration of the intervention and baseline values, the significant differences in HbA_1c_ and ISI between the groups disappeared. Furthermore, after intervention, on the basis of HbA_1c_ IFG or IGT, no significant remission and progression from prediabetes to diabetes were found between the intervention and NI groups (Table 2).

### Intention to treat (ITT) analysis

When using ITT analysis, the significant changes of HFC, body weight, FM and fat% of the whole body as well as FM in the abdominal region in all intervention groups remained but no group by time differences were found (Table 3). However, after controlling for the change of body weight, duration of the intervention and baseline values, significant group differences were found for HFC (AEx vs. NI p = 0.006, Diet vs. NI p = 0.006, AED vs. NI p < 0.001, respectively), FM (AEx vs. NI p = 0.011, Diet vs. NI p = 0.048, AED vs. NI p = 0.013, respectively), fat% (AEx vs. NI p = 0.003, Diet vs. NI p = 0.042, AED vs. NI p = 0.010, respectively). Significant changes were also found between AED and NI groups in body weight (p = 0.005), VAT (p = 0.008) and FM_android_ (p = 0.025) after controlling for the change of body weight, duration of the intervention and baseline values. The effect size was η = 0.097 (p = 0.010) for HFC, η = 0.116 (p = 0.003) for FM, η = 0.128 (p = 0.002) for fat%, η = 0.078 (p = 0.029) for FM_android_, and η = 0.066 (p = 0.054) for VAT, respectively. However, the significant changes in HbA_1c_ and insulin sensitivity found in the efficacy analysis disappeared, and no significant group by time difference was found in fasting and two-hour glucose and insulin, serum triglycerides, free fatty acids FFA, and liver enzymes (Table 3).

**Table 3:** Mean (95% confidence interval) values of HFC, body composition, biomarkers during intervention over 6-month period (ITT).

Variables	NI (1)		AEx (2)		Diet (3)		AED (4)		Group by time									
	Baseline	Inter-vention	Baseline	Inter-vention	Baseline	Inter-vention	Baseline	Inter-vention	Within Groups				Between Groups					
									NI	AEx	Diet	AED	1-2	1-3	1-4	2-3	2-4	3-4
HFC (%)	16.0 (12–20)	18.8 (15–23)	17.7 (13–22)	12.2 (8–16)	16.7 (12–21)	11.3 (7–15)	17.2 (13–22)	9.6 (6–14)	0.222	0.018	0.025	0.001	0.318	0.159	0.105	0.763	0.530	0.842
Weight (kg)	69.5 (66–73)	69.7 (66–74)	72.3 (69–76)	71.3 (67–75)	68.7 (65–73)	67.9 (64–72)	70.3 (67–74)	68.8 (65–73)	0.795	0.046	0.084	0.003	0.423	0.645	0.995	0.211	0.419	0.649
LM (kg)	40.6 (38–43)	40.4 (38–43)	42.5 (40–45)	42.5 (40–45)	41.0 (38–44)	41.0 (38–44)	42.3 (40–45)	42.0 (39–45)	0.478	0.960	0.973	0.185	0.296	0.807	0.398	0.428	0.841	0.553
FM_WB_ (kg)	26.3 (24–28)	26.5 (25–29)	27.4 (25–29)	26.2 (24–28)	25.6 (24–28)	25.0 (23–27)	25.8 (24–28)	24.6 (23–27)	0.432	<0.001	0.072	<0.001	0.785	0.415	0.372	0.279	0.245	0.944
Fat (%)	38.0 (36–40)	38.3 (36–40)	38.1 (36–40)	37.0 (35–39)	37.3 (36–39)	36.9 (35–39)	36.7 (35–39)	35.8 (34–38)	0.253	<0.001	0.078	0.001	0.682	0.443	0.166	0.717	0.328	0.543
FM_Android_ (kg)	2.7 (2.5–3.0)	2.8 (2.5–3.0)	2.7 (2.5–3.0)	2.6 (2.4–2.9)	2.6 (2.3–2.8)	2.5 (2.2–2.7)	2.5 (2.3–2.8)	2.3 (2.1–2.6)	0.454	0.019	0.121	0.001	0.653	0.188	0.58	0.382	0.146	0.568
FM_Gynoid_ (kg)	3.4 (3.1–3.8)	3.4 (3.1–3.7)	3.7 (3.3–4.0)	3.5 (3.2–3.8)	3.4 (3.0–3.7)	3.3 (3.0–3.6)	3.4 (3.1–3.8)	3.3 (2.9–3.6)	0.619	0.001	0.177	0.005	0.476	0.752	0.788	0.307	0.327	0.960
VAT (kg)	1.8 (1.5–2.1)	1.9 (1.5–2.2)	1.7 (1.4–2.0)	1.6 (1.2–1.9)	1.6 (1.3–1.9)	1.3 (1.0–1.7)	1.7 (1.4–2.0)	1.3 (1.0–1.6)	0.520	0.223	0.098	0.001	0.382	0.064	0.102	0.321	0.443	0.815
SAT (kg)	2.3 (1.9–2.6)	2.2 (1.8–2.6)	2.6 (2.3–2.9)	2.4 (2.1–2.8)	2.2 (1.8–2.5)	2.1 (1.7–2.5)	2.2 (1.8–2.5)	2.0 (1.6–2.3)	0.444	0.040	0.272	0.014	0.238	0.697	0.500	0.120	0.065	0.780
Glucose fasting (mmol/L)	5.6 (5.4–5.9)	5.8 (5.6–6.1)	5.7 (5.4–5.9)	5.5 (5.3–5.8)	5.2 (5.0–5.5)	5.5 (5.3–5.8)	5.5 (5.3–5.8)	5.6 (5.4–5.9)	0.132	0.458	0.042	0.338	0.406	0.025	0.369	0.149	0.946	0.169
Glucose 2-h (mmol/L)	8.2 (7.6–8.8)	8.1 (7.1–91)	8.1 (7.4–8.7)	7.7 (6.7–8.6)	7.8 (7.2–8.5)	8.1 (7.1–9.1)	8.4 (7.7–9.0)	8.2 (7.2–9.2)	0.869	0.477	0.605	0.780	0.486	0.669	0.750	0.792	0.310	0.457
Insulin fasting (µIU/mL)	15.7 (12–20)	15.8 (13–18)	16.2 (13–19)	16.1 (14–19)	14.4 (11–18)	14.3 (12–17)	16.5 (13–20)	15.8 (13–18)	0.675	0.922	0.939	0.651	0.668	0.542	0.691	0.302	0.975	0.316
Insulin 2-h (µIU/mL)	120 (91–149)	100 (70–131)	107 (78–137)	87 (57–117)	118 (89–147)	79 (57–102)	123 (94–153)	90 (68–112)	0.275	0.264	0.036	0.071	0.328	0.391	0.801	0.910	0.473	0.548
HbA_1c_ (%)	6.2 (6.0–6.3)	6.2 (6.0–6.3)	6.1 (6.0–6.3)	6.1 (6.0–6.3)	6.1 (5.9–6.2)	6.0 (5.9–6.2)	6.1 (5.9–6.3)	6.0 (5.8–6.2)	0.778	0.911	0.873	0.251	0.669	0.234	0.274	0.442	0.504	0.915
ISI	62 (50–74)	65 (52–79)	66 (53–78)	75 (62–88)	74 (61–87)	79 (65–92)	59 (47–72)	72 (59–85)	0.646	0.187	0.525	0.081	0.361	0.097	0.797	0.446	0.507	0.197
Triglyceride (mmol/L)	2.3 (1.8–2.9)	2.2 (1.7–2.7)	1.9 (1.4–2.5)	2.0 (1.5–2.5)	2.1 (1.5–2.6)	2.1 (1.6–2.6)	2.2 (1.6–2.7)	2.3 (1.8–2.8)	0.568	0.766	0.946	0.590	0.324	0.528	0.957	0.729	0.351	0.563
FFA (mmol/L)	0.6 (0.5–0.6)	0.5 (0.4–0.6)	0.6 (0.5–0.7)	0.4 (0.4–0.5)	0.6 (0.5–0.7)	0.5 (0.4–0.5)	0.5 (0.4–0.6)	0.5 (0.4–0.6)	0.173	0.001	0.003	0.522	0.849	0.633	0.842	0.499	0.993	0.495
ALT (U/L)	22.0 (17–27)	23.5 (19–28)	22.8 (18–28)	21.3 (17–26)	22.6 (18–28)	18.2 (14–23)	23.6 (19–29)	22.1 (18–27)	0.638	0.641	0.168	0.627	0.795	0.376	0.962	0.530	0.759	0.352
AST (U/L)	23.4 (20–27)	27.4 (24–31)	26.9 (23–30)	23.7 (20–27)	22.5 (19–26)	22.9 (19–26)	26.8 (23–30)	21.9 (18–25)	0.091	0.173	0.866	0.034	0.978	0.169	0.593	0.174	0.609	0.390
GGT (U/L)	33.3 (29–38)	30.7 (25–37)	26.2 (21–31)	27.4 (21–34)	22.5 (18–27)	24.3 (18–31)	27.6 (23–32)	27.9 (22–34)	0.285	0.603	0.470	0.896	0.142	0.017	0.226	0.341	0.794	0.227

Mixed model for repeated measures (2 factor interactions: group × time) followed by Sidak correction for within and between groups. Data were imputed to include all subjects who were randomized to different intervention groups (n = 115).

ITT = Intention to treatment analysis; NI = No intervention group; AEx = Exercise group; AED = AEx + Diet group; FM = Fat mass, HFC = Hepatic fat content; LM = Lean mass, VAT = abdominal visceral fat content, SAT = abdominal subcutaneous fat content, ISI = insulin sensitivity index (Matsuda Index); HbA_1c_ = glycated haemoglobin; FFA = Free fatty acids, ALT = Alanine aminotransferase; AST = aspartate aminotransferase; GGT = γ-glutamyltransferase.

### Other outcomes

Aerobic fitness (VO_2max_) increased in all groups but the increase was greater in the AED and AEx groups compared to the NI group (p < 0.05 for both) 0.001) (Table S1). There were no significant group by time differences in the dietary energy and energy yield nutrient intakes, except for fibre (Table S1), which was increased in the diet and AED groups compared to the AEx and NI groups (p = 0.006 −<0.01), with an effect size (η) of 0.38.

## Discussion

The current study shows, for the first time according to our knowledge that combined aerobic exercise and fibre-enriched diet reduces hepatic steatosis more than either exercise or increased fibre intake alone. In fact, HFC decreased to normal level (<5.6%) in 44% of the participants in the exercise plus diet group. These findings have significant clinical implications for pre-diabetic patients with NAFLD because they show that if lifestyle interventions are instituted at the appropriate time they can prevent NAFLD progression.

In China the prevalence of NAFLD and prediabetes is increasing rapidly, reaching epidemic proportions^2^. These metabolic disorders are largely attributable to lifestyle changes that have occurred over recent decades, and therefore specific lifestyle interventions attempting to modify patients’ diet and physical activity may have the potential to reduce the severity of NAFLD and prevent development of diabetes^14,15^. Vilar-Gomez *et al*.^16^ demonstrated that level of improvement in histologic features of nonalcoholic steatohepatitis (NASH) after lifestyle intervention is associated with the degree of weight loss. A recent meta-analysis of twenty randomized controlled trails with 1073 NAFLD patients found that exercise can exert beneficial effects on intrahepatic triglycerides even in the absence of weight loss^15^. Our study complements this by showing significant reduction in HFC in the intervention groups, independent of change in body weight.

Previous studies indicate that patients affected with NAFLD are at increased risk of developing inflammation and subsequently fibrosis and chronic liver disease^17,18^. In order to obtain accurate information regarding the degree of inflammation and the present of fibrosis, repeated liver biopsies would have been needed. However, given the invasive nature of the biopsy procedure and the possible risks it pertains, it would have been unethical to obtain biopsies from the “healthy” patients in the present study. In fact, our aim was not to study NASH; instead we focused on the most sensitive non-invasive parameter (liver fat content measured with ^1^H MRS) which has been shown to be highly correlated with whole body insulin sensitivity^19^ and metabolic imbalance can be detected at an early stage (liver fat content varying between 0.5% and 39.3%)^20^. This illustrating ^1^H MRS provides unique information which cannot be obtained otherwise.

In the Da Qing study^21^ exercise training and/or diet counselling led to a significant decrease in the incidence of diabetes over a 6-year period among those with impaired glucose tolerance. The Finnish Diabetes Prevention Study showed that, in addition to physical activity, increased fibre intake was associated with long-term weight loss and decreased T2D risk^22^. Subsequently, randomized controlled trials have shown that increased fibre intake improves glycaemic control in patients with T2D^9^. However, no previous study has assessed the combined effects of aerobic exercise training and fibre-enriched diet on glycaemic control and HFC reduction in patients with prediabetes and NALFD. Here, we show that combined exercise and fibre-enriched diet decreased HFC more than either intervention mode alone. Unexpectedly however, no significant improvement in glycaemic control was found, suggesting dissociation between hepatic steatosis and insulin resistance. This finding is in agreement with animal and human data, which have shown that under certain conditions simple hepatic steatosis is not accompanied by metabolic dysregulation but is associated with a metabolically benign state^23,24^. Thus, it could be that hepatic fat infiltration might represent an adaptive mechanism allowing storage of lipids in their least toxic form in situations where triglyceride precursors are abundant. However, when hepatic fat infiltration reaches a critical point, the compensatory mechanism becomes overwhelmed resulting in damaged hepatocytes and impairment of glucose and lipid metabolism^24^. Our results are therefore important from the clinical point of view because simple NAFLD can develop into more severe forms of liver disease and with existing pre-diabetes can increase the risk for T2D and cardiovascular diseases^4^. Thus, our results suggest that increased fibre intake could be used as adjuvant to exercise training in patients with such comorbid conditions to prevent disease progression. More randomized controlled studies with larger samples size are needed to confirm these findings.

In earlier intervention studies exercise training has generally been performed by using brisk walking or treadmill^25,26^. In the present study, patients exercised using Nordic walking, which is a walking technique using two poles and mimicking the movements performed while cross-country skiing. This walking technique is associated with the involvement of more muscle groups than in the case of classic walking, and therefore makes it possible to increase exercise intensity, resulting in more effective training in a safe and well tolerated way^27^. Nordic walking has shown to be effective in reducing fat mass^28^. Besides exercise, increased soluble fibre intake may have played an important role in reducing fat mass and increasing insulin sensitivity in the present study. Our results suggest when the fibre intake is low, increasing the fibre intake even to the recommended level may have significant impact on health. Previous work has shown that soluble fibre modulates the postprandial glycaemic response through its effects on the stomach and small bowel^9^. In addition, both soluble and insoluble fibre intake can improve glycaemic control by increasing insulin sensitivity^29^. However, the mechanisms associated with the beneficial effect of fibre-enriched diet on glycaemic control have not yet been fully established and the basis of the effect on HFC reduction is even more unclear. Nonetheless, this finding is all the more important for pre-diabetic patients with NAFLD who have heightened risk for type 2 diabetes.

Nutrition and meal planning are known often to be the most challenging aspects of diabetes self-care^30^. The novelty of this study is that, in addition to dietary counselling, we provided lunch to both diet and exercise plus diet intervention groups for the entire duration of the intervention. Thus the daily nutritional intake at least one-third of the meals of each subject was controlled, which is likely to increase the reliability of our results. In addition, the exercise training was performed in community parks near the participants’ home to make attendance of the supervised sessions as convenient as possible, thereby increasing compliance. However, because all participating subjects originated from the same community, the behaviour of subjects in the no-intervention group may have been affected by interaction with subjects in the intervention groups, even though we asked the no intervention subjects to maintain their existing lifestyle during the intervention. This is, for example, reflected by the observation that the fitness level of the no-intervention group also increased. It is important to note that there were no significant differences in total energy intake and energy yield nutrients between groups, except for the increased intake of fibre in the diet and diet plus exercise groups. Finally, an important aspect of the current study is that it was conducted within a large community, meaning the district volunteers, the nurses from health care centres, hospital doctors and university researchers all had to work seamlessly together to make this study possible. Thus, this study presents a model that can be applied through a community’s health education and promotion program which can result in significant health benefits for its participants. We feel that all the aforementioned aspects aiming to optimally control the current study add to the reliability of the observed results and strengthen our conclusions.

We recognize that the sample size in our study is relatively small but the observed effect sizes are similar to other intervention studies reported in systematic reviews for randomized lifestyle trials^10,12^. Of note, the main outcomes of the intervention have been assessed with both efficacy and intention-to treat analysis. Recently, a variation in the PNPLA3 gene has been reported to influence the development of NAFLD^31^. However, in our study, there were no significant differences in the distribution of PNPLA3 genotypes (GG, GC and CC) among the groups and thus response of exercise and diet intervention was unlikely to be affected by this gene variant. Giving the abovementioned reasons, the significant results obtained in this study are conservative and justified. However, studies with larger sample sizes are needed to confirm our findings.

In summary, people who have both prediabetes and NAFLD are at much higher risk of developing type 2 diabetes and cardiovascular diseases^32,33^, and therefore finding effective ways to combat this comorbid condition are urgently needed. In this study we demonstrated that aerobic exercise training combined with fibre-enriched diet can help to reduce hepatic fat content in pre-diabetic patients with NAFLD better than either increased exercise or fibre intake alone.

## Methods

### Trial design and study population

This study was a four-arm parallel-group, randomized controlled trial, conducted in 7 health clinical service centres in the Shanghai Yangpu district. More detailed information regarding study design and subjects are given in our protocol paper^34^. This report is a part of the original study design and the modification made for this report is given in supplement 1. The study was approved by the Ethics Committee of Shanghai Institute of Nutrition (06.01.2013, ref: 2013-003) and has been registered in the International Standard Randomized Controlled Trial Number Register (ISRCTN 42622771). All participants provided written informed consent prior to the study. The study was followed the world medical association declaration of Helsinki – Ethical principles for medical research involving human subjects.

The eligibility criteria for intervention groups were as follows: Men or women aged 50–65 years with impaired fasting glucose (IFG between 5.6 to 6.9 mmol/L) or impaired glucose tolerance (IGT between 7.8 to 11.0 mmol/L 2 hour after the intake of 75 g glucose), and diagnosed as NAFLD by ^1^H MRS (liver fat > 5%)^35^ and by questionnaire that on-going or recent alcohol consumption is <21 drinks on average per week in men and <14 drinks on average per week in women^36^; no chronic cardiovascular, serious musculoskeletal or gastrointestinal problems and not on extreme diets; and for women, serum follicle-stimulating hormone level greater than 30 IU/L and last menstruation more than 6 months ago but within 10 years. The exclusion criteria included body mass index (BMI) >38 kg/m^2^; serious cardiovascular or musculoskeletal problems; diagnosed with Type 1 diabetes and T2D; and mental illness.

### Randomisation and blinding

After an initial screening, potential subjects were assessed for inclusion in the run-in period of the trial. The subjects were given detailed information regarding the study through information meeting in the health care centres. After eligibility was confirmed, we randomly assigned (1:1:1:1) participants to aerobic exercise (AEx, n = 29), diet intervention (Diet, n = 28), aerobic exercise plus diet intervention (AED = 29), or no intervention (NI = 29) groups for minimal 6 months. A computer program was used to generate the block randomisation sequence (block size 20) and was controlled by a researcher not involved in the selection of the participants. Although lifestyle interventions cannot be performed in a double-blinded fashion (since study subjects clearly are aware of the type of intervention), investigators were blinded for the tests and analyses during the entire study.

### Study settings

The laboratory tests and interventions were performed and managed by the research team from Shanghai University of Sport, Shanghai Jiao Tong University, Shanghai Yangpu District Health Care Service Centres and Yungpu Shidong Hospital, and Clinical Nutrition Centre at Fudan University Huadong Hospital, Shanghai, China.

### Procedures

The specific design of this particular study was recently published in a detailed protocol paper by our group^34^. Briefly, potential subjects were selected from the outpatient pool with both a liver ultrasound (for diagnosing fatty liver) and fasting blood results (for diagnosing diabetes) on file during the period of 2012 to 2013. After signing the screening consent, patients were asked to fill in a screening questionnaire to assess their health and medication history, including physical activity and alcohol consumption, and to undergo a glucose tolerance test. Those subjects who met the inclusion criteria and were willing to participate in this study were invited for measurements of height, weight, waist circumference, blood pressure, physical performance, and body composition by dual-X-ray densitometry (DXA, GE, USA, Prodigy). They were also invited for a proton magnetic resonance spectroscopy (^1^H MRS) scan for non-invasive assessment of liver fat, with NAFLD defined as >5% liver fat content (Siemens Magnetom Verio 3 T, Siemens AG, Erlangen, Germany). The qualifying subjects were then randomized into different groups and attended information meetings organized for each individual group after which the intervention started. The baseline measurement of the HFC to the intervention starting day was within 10 days. At 1 week after intervention was stopped, the HFC was assessed. The average duration of the intervention was 8.6 months ranged from 6.8 to 11 months.

*The exercise (AEx) group*, after baseline fitness assessments, participated 2-3 times a week in a supervised progressive aerobic exercise training program (such as Nordic brisk walking + stretching and other group exercises) which was developed by an exercise researcher. The exercise sessions were performed at the community park areas which were close to the subjects’ home. The intensity and duration of exercise was increased from 60% to 75% of the maximum oxygen uptake (estimated from fitness test) and from 30 to 60 min per session. In addition, each exercise sessions included a 5 min warm-up and 5 min cool-down period (such as stretching and group exercise).

*The diet group*, after baseline assessments, had a daily lunch plus an individual nutritional consultation program developed by a clinical nutritionist on the basis of each individual’s dietary intakes and body weight. During the intervention, subjects were given a daily prepared meal (lunch), which accounted for 30–40% of the total daily energy intake. The meal included 37–40% carbohydrate with 9–13 g as fibre, 35–37% fat (SAFA 10%, MUFA 15–20%, PUFA 10%) and 25–27% protein. To ensure subjects consumed sufficient amounts of dietary fibre, 5 g of soluble fibre (dietary water soluble fibre) was also added to the lunch.

The meals were prepared at the canteen of Shanghai University of Sport under the guidance of a clinical nutritionist. Each meal contained three to four dishes of foods commonly eaten by Chinese in Shanghai. A trained study staff member weighed each item for each specific person according to the dietary plan and the cooked dishes were put into a named lunch box for each participant. The lunch box was then delivered to the study district community office where the study subjects were gathered. If the participants did not have time to come to pick up the meal, it was delivered to their home and the participants ate it for dinner. The other two meals (breakfast and dinner) were cooked by subjects themselves following the nutritionist advice. In addition, the diet group was advised to maintain their current level of physical activity during the intervention.

*The Exercise plus Diet (AED) group* performed the same exercise program and followed the same diet as described above for exercise and diet groups.

*The no intervention (NI) group* was advised to maintain their current level of physical activity and eating habits during the intervention. After intervention, the control group was provided with an opportunity to participate in our exercise and diet intervention program for 3 months. In this report, the follow-up results of these subjects are not included.

## Measurements

All the measurements were performed at the baseline and minimal 6 months after intervention at the Shanghai Sport University laboratory.

*The primary endpoint variable* was the change of HFC. Non-invasive measurement of HFC was performed using single-voxel proton magnetic resonance spectroscopy (^1^H MRS) on a 3 T Siemens Magnetom Verio scanner (Siemens Medical Solutions, Erlangen, Germany). Obtained spectra were analysed using the Linear Combination of Model spectra software suite which is generally considered to be the gold standard for *in-vivo* spectroscopy analysis^35^. Briefly, the methylene and methyl peak amplitudes of the fat spectrum and the amplitude of the water spectrum were corrected because of differences in T2 decay^37^ and molar concentrations of ^1^H nuclei in fat and water^38^. HFC was defined as fat in relation to the total weight of liver tissue and was calculated with the following equation: HFC = Sf/[Sf + (Sw/0.7)], where Sf and Sw indicate the amplitudes of fat and water in the spectrum, respectively^37^.

### The other key assessments including

Abdominal subcutaneous (SAT) and visceral (VAT), retroperitoneal (RAT) and intraperitoneal (IAT) fat of total region from the tip of the xyphoid to L5 was measured by ^1^H MRS. Measured volumes of the abdominal adipose tissue compartments were converted into tissue mass (kg) taking into account slice thickness and an adipose tissue density of 0.9196 g/ml^39^. In this report, only SAT and VAT results are included.

Glucose, insulin, insulin sensitivity index of whole-body (ISI = 10,000/square root of [fasting glucose x fasting insulin] x [mean glucose x mean insulin during OGTT]^40^ were assessed from glucose tolerance test (performed after overnight fasting, 30 minutes and 2 hours after the intake of 75 g glucose). In addition, glycated haemoglobin (HbA_1c_), total cholesterol (TC), triglycerides, alanine aminotransferase (ALT); aspartate aminotransferase (AST); γ-glutamyl transferase (GGT); were measured from serum sample by conventional methods.

rs738409, a DNA sequence variant (single nucleotide polymorphism) located in the patatin-like phospholipase domain-containing protein 3 **(**PNPLA3) locus, was assessed by forward 5′-atggggagcaaggagaggaa-3′ and reverse 5′-cgggtagcctggaaataggg-3′31. The DNA fragments were amplified with the 2X Taq PCR Master Mix (Lifefeng Biotech). Direct sequencing of PCR products was performed using the ABI Prism Big Dye Terminator v3.1 Cycle Sequencing Kit (Applied Biosystems) on an ABI Prism 3700 DNA Analyzer (Applied Biosystems). All sequences were analysed by the Chromas Software (Technelysium)^31^.

2 km walking test was performed by walking 2 kilometres as fast as possible on a flat surface^41^. The result was recorded as a fitness index taking into account person’s age, gender, height, weight, time taken to walk 2 kilometres and the heart rate at the end of the test. There were five fitness classes which can be used to compare result with the fitness of others of the same age and gender or a person’s own previous results and development between the tests. The individual’s result was used to set up their own personalized exercise program.

The dietary results are calculated on the basis of the China food composition^42^. In this book, the calculation of fibre is only included insoluble fibre. The results of fibre for the diet and AED groups after intervention included also added 5 g of soluble fibre.

### Sample size

In the initial study design, we estimated the sample size based on previous literature of liver fat and microbiota as primary outcome variable. We estimated that 34 subjects in each group would have 85% power for mean comparison between the groups and therefore we attempted targeting to have 50 subjects in each group. However, during the recruitment period, we found that only about 20% of the subjects met the inclusion criteria and due to the limited funding, we re-calculated the sample size based on of our previous similar type of study result (changing in fat mass). Thus, for this report, sample size estimation for the primary outcome HFC with 29 individuals has 95% power to test against the hypothesis that there is change in any group. Further, when intervention groups compared with NI group having 17 subjects, the power for the HFC was 84% with <0.05 two sided significance level. After every subject reached minimal 6 months intervention, the study did not continue to follow-up all the subjects and consequently, the follow-up study was terminated.

### Statistical analysis

All data were analyzed by PASW statistics version 22 (IBM Corporation, USA). If data were not normally distributed, their natural logarithms were used. Descriptive statistics were used to present the background and anthropometric data at the baseline and follow-up assessments as mean and standard deviation (SD) unless otherwise stated.

An efficacy analysis was performed to compare the percentage change among the groups. If a participant in the AEx and AED groups did not exercise for more than 4 weeks, or a participant in the Diet group did not take the lunch provided for 4 weeks due to different reasons, they were not included in this analysis. The number of participants in this analysis was 18 in the NI, 19 in the AEx, 21 in the Diet and 20 in the AED groups, respectively.

Mixed model for the repeated measure in an intention-to-treat (ITT) analysis was performed to compare the AEx, Diet and AED groups to the NI group. To be able to perform the ITT analysis by using all the subjects randomized into different groups (n = 115), we used linear regression to impute the missing data. The interaction of group by time was compared by contrast to the NI group and within a group change was compared by time. In addition, change of body weight, intervention duration and baseline value were used as covariates. If the significance of the group by time interaction was p < 0·05, the effect was estimated utilizing Šidák for multiple comparisons.

The percentage differences were calculated for the period between baseline and end point measurements for each individual. The comparison of percentage changes in different groups were performed using ANCOVA (treatment group × time) controlled for the baseline value using Šidák for multiple comparisons.

Measures of effect size in the ANCOVA and repeated-measures ANOVA were shown in partial η^2^. Partial η^2^ values that reached 0.01, 0.06, and 0.14 were regarded as small, medium, and large effect sizes, respectively^43^. All tests were two-tailed, and a p value of less than 0.05 was set as significant.

## Electronic supplementary material

Supplement 1

Supplement Table S1

## References

[1] Angulo P (2007). GI epidemiology: nonalcoholic fatty liver disease. Aliment Pharmacol Ther.

[2] Fan JG (2013). Epidemiology of alcoholic and nonalcoholic fatty liver disease in China. J Gastroenterol Hepatol.

[3] Xu Y (2013). Prevalence and control of diabetes in Chinese adults. Jama.

[4] Lonardo A, Ballestri S, Marchesini G, Angulo P, Loria P (2015). Nonalcoholic fatty liver disease: a precursor of the metabolic syndrome. Dig Liver Dis.

[5] Swinburn BA, Metcalf PA, Ley SJ (2001). Long-term (5-year) effects of a reduced-fat diet intervention in individuals with glucose intolerance. Diabetes care.

[6] Whitsett M, VanWagner LB (2015). Physical activity as a treatment of non-alcoholic fatty liver disease: A systematic review. World J Hepatol.

[7] Houmard JA (2004). Effect of the volume and intensity of exercise training on insulin sensitivity. J Appl Physiol (1985).

[8] Chalasani N (2012). The diagnosis and management of non-alcoholic fatty liver disease: practice Guideline by the American Association for the Study of Liver Diseases, American College of Gastroenterology, and the American Gastroenterological Association. Hepatology.

[9] Silva FM (2013). Fiber intake and glycemic control in patients with type 2 diabetes mellitus: a systematic review with meta-analysis of randomized controlled trials. Nutr Rev.

[10] Yoon U, Kwok LL, Magkidis A (2013). Efficacy of lifestyle interventions in reducing diabetes incidence in patients with impaired glucose tolerance: a systematic review of randomized controlled trials. Metabolism.

[11] Rohling, M., Herder, C., Roden, M., Stemper, T. & Mussig, K. Effects of Long-Term Exercise Interventions on Glycaemic Control in Type 1 and Type 2Diabetes: a Systematic Review. *Experimental and clinical endocrinology & diabetes: official journal, German Society of Endocrinology [and] German Diabetes Association* (2016). 10.1055/s-0042-106293 27437921

[12] Golabi P (2016). Effectiveness of exercise in hepatic fat mobilization in non-alcoholic fatty liver disease: Systematic review. World J Gastroenterol.

[13] Orci LA (2016). Exercise-based Interventions for Nonalcoholic Fatty Liver Disease: A Meta-analysis and Meta-regression. Clinical gastroenterology and hepatology: the official clinical practice journal of the American Gastroenterological Association.

[14] Romero-Gomez, M., Zelber-Sagi, S. & Trenell, M. Treatment of NAFLD with diet, physical activity and exercise. *J Hepatol* (2017). 10.1016/j.jhep.2017.05.016 28545937

[15] Katsagoni CN, Georgoulis M, Papatheodoridis GV, Panagiotakos DB, Kontogianni MD (2017). Effects of lifestyle interventions on clinical characteristics of patients with non-alcoholic fatty liver disease: A meta-analysis. Metabolism.

[16] Vilar-Gomez, E. *et al*. Weight Loss Through Lifestyle Modification Significantly Reduces Features of Nonalcoholic Steatohepatitis. *Gastroenterolog*y **149** (2), 367–378 e365; quize314–365 (2015). 10.1053/j.gastro.2015.04.005 25865049

[17] Browning JD (2004). Prevalence of hepatic steatosis in an urban population in the United States: impact of ethnicity. Hepatology.

[18] Adams LA, Lindor KD (2007). Nonalcoholic fatty liver disease. Ann Epidemiol.

[19] Borra R (2008). Inverse association between liver fat content and hepatic glucose uptake in patients with type 2 diabetes mellitus. Metabolism.

[20] Machann J (2006). Hepatic lipid accumulation in healthy subjects: a comparative study using spectral fat-selective MRI and volume-localized 1H-MR spectroscopy. Magn Reson Med.

[21] Pan XR (1997). Effects of diet and exercise in preventing NIDDM in people with impaired glucose tolerance. The Da Qing IGT and Diabetes Study. Diabetes care.

[22] Lindstrom J (2006). High-fibre, low-fat diet predicts long-term weight loss and decreased type 2 diabetes risk: the Finnish Diabetes Prevention Study. Diabetologia.

[23] Monetti M (2007). Dissociation of hepatic steatosis and insulin resistance in mice overexpressing DGAT in the liver. Cell Metab.

[24] Stefan N, Haring HU (2011). The metabolically benign and malignant fatty liver. Diabetes.

[25] Praet SF (2008). Brisk walking compared with an individualised medical fitness programme for patients with type 2 diabetes: a randomised controlled trial. Diabetologia.

[26] Rezende, R.E. *et al*. Randomized clinical trial: benefits of aerobic physical activity for 24 weeks in postmenopausal women with nonalcoholic fatty liver disease. *Menopause* **23** (8), 876–883. 10.1097/GME.0000000000000647 27458060

[27] Tschentscher, M., Niederseer, D. & Niebauer, J. Health benefits of Nordic walking: a systematic review. *Am J Prev Med* **44** (1), 76–84. 10.1016/j.amepre.2012.09.043 23253654

[28] Gram, B., Christensen, R., Christiansen, C. & Gram, J. Effects of nordic walking and exercise in type 2 diabetes mellitus: a randomized controlled trial. *Clin J Sport Med* **20** (5), 355–361. 10.1227/NEU.0b013e3181e56e0a 20818193

[29] Ylonen K (2003). Associations of dietary fiber with glucose metabolism in nondiabetic relatives of subjects with type 2 diabetes: the Botnia Dietary Study. Diabetes care.

[30] Campbell AP, Rains TM (2015). Dietary protein is important in the practical management of prediabetes and type 2 diabetes. J Nutr.

[31] Luukkonen PK (2016). Hepatic ceramides dissociate steatosis and insulin resistance in patients with non-alcoholic fatty liver disease. J Hepatol.

[32] Williamson RM (2011). Prevalence of and Risk Factors for Hepatic Steatosis and Nonalcoholic Fatty Liver Disease in People With Type 2 Diabetes: the Edinburgh Type 2 Diabetes Study. Diabetes care.

[33] Stefan, N., Fritsche, A., Schick, F. & Haring, H. U. Phenotypes of prediabetes and stratification of cardiometabolic risk. The lancet. Diabetes & endocrinology **4** (9), 789–798. 10.1016/S2213-8587(16)00082-6 27185609

[34] Liu WY (2014). Effect of aerobic exercise and low carbohydrate diet on pre-diabetic non-alcoholic fatty liver disease in postmenopausal women and middle aged men - the role of gut microbiota composition: study protocol for the AELC randomized controlled trial. BMC Public Health.

[35] Borra RJ (2009). Nonalcoholic fatty liver disease: rapid evaluation of liver fat content with in-phase and out-of-phase MR imaging. Radiology.

[36] Sanyal AJ (2011). Endpoints and clinical trial design for nonalcoholic steatohepatitis. Hepatology.

[37] Thomsen C (1994). Quantification of liver fat using magnetic resonance spectroscopy. Magn Reson Imaging.

[38] Szczepaniak LS (1999). Measurement of intracellular triglyceride stores by H spectroscopy: validation *in vivo*. Am J Physiol.

[39] Provencher SW (2001). Automatic quantitation of localized *in vivo* 1H spectra with LCModel. NMR Biomed.

[40] Matsuda M, DeFronzo RA (1999). Insulin sensitivity indices obtained from oral glucose tolerance testing: comparison with the euglycemic insulin clamp. Diabetes care.

[41] Laukkanen R, Oja P, Pasanen M, Vuori I (1992). Validity of a two kilometre walking test for estimating maximal aerobic power in overweight adults. Int J Obes Relat Metab Disord.

[42] Yuexin, Y., Guangya, W. & Xingchang, P. *China food composition* (Peking University Medical Press, National Institute of Nutrition and Food Safety, China CDC, 2011).

[43] Hillsdale, N. & Erlbaum. *Statistical power analysis for the behavioral sciences* (1988).

