# Supplementary material for: Effect of aerobic exercise and diet on liver fat in pre-diabetic patients with non-alcoholic-fatty-liver-disease: A randomized controlled trial
Source: Sci Rep. 2017 Nov 21;7:15952. doi: 10.1038/s41598-017-16159-x (PMC5698376; doi:10.1038/s41598-017-16159-x)
Supplement: Supplementary file 1 — Supplement 1 [file 41598_2017_16159_MOESM1_ESM.pdf]

Effect of aerobic exercise and diet on liver fat in pre-diabetic patients with non-alcoholic-fatty-liver-disease: A randomized controlled trial

Authors

Sulin Cheng, PhD[1,4,5*], Jun Ge, MD[2*], Can Zhao, PhD[3], Shenglong Le, M.Sc[1], Yifan Yang, M.Sc[1], Dandan Ke[3], Na Wu, M.Sc[1], Xiao Tan, PhD[1,4], Xiaobo Zhang M.Sc[1], Xiamin Du MD[2], Jianqin Sun, PhD[6], Renwei Wang, PhD[3], Yongyong Shi PhD[7], Ronald J. H. Borra, MD, PhD[8,9], Riitta Parkkola, MD, PhD[8], Petri Wiklund, PhD[10,11,4], Dajiang Lu M.Sc[3*]

Modification made in original study protocol

After publishing our original study protocol[1], some modifications have been made and updated in the International Standard Randomized Controlled Trial Number Register (ISRCTN 42622771). The modifications are mainly related to sample size estimation. In the initial study design, we estimated the sample size based on previous literature of liver fat and microbiota as primary outcome variable. We estimated that 34 subjects in each group would have 85% power for mean comparison between the groups and therefore we targeted to have 50 subjects in each group. However, during the recruitment period, we found that only about 20% of the subjects met the inclusion criteria and due to the limited funding and time, we had to re-calculate the sample size based on results of our previous study (changing in fat mass). Thus, for this report, sample size estimation for the primary outcome HFC with 29 individuals has 95 % power to test against the hypothesis that there is no change in any group. Further, when intervention groups compared with NI group having 17 subjects, the power for the HFC was 84 % with <0.05 two sided significance level. After minimal 6 months intervention, the follow-up study was terminated.

Another primary outcome of microbiota is still under analysis; therefore, the results are not included in this report.

In addition, in the statistical analyses, to avoid the effect of different variances in different groups on the outcomes; we have used Mixed model in stand of analysis of variance (ANOVA

or ANCOVA) for the repeated measure in ITT analysis. The interaction of group by time was compared by contrast to the no intervention group and within a group change was compared by time. The Bonferroni for multiple comparisons have changed to Šidák which is consulted by a statistician.

Measures of effect size were added in this report to ensure that the results are not biased by sample. Measures of effect size in the analyses were shown in partial η2. Partial η2 values that reached 0.01, 0.06, and 0.14 were regarded as small, medium, and large effect sizes, respectively[2].

The other key assessments including:

rs738409, a DNA sequence variant (single nucleotide polymorphism) located in the patatin-like phospholipase domain-containing protein 3 **(**PNPLA3) locus, was assessed by forward 5'-atggggagcaaggagaggaa-3' and reverse 5'-cgggtagcctggaaataggg-3'3. The DNA fragments were amplified with the 2X Taq PCR Master Mix (Lifefeng Biotech). Direct sequencing of PCR products was performed using the ABI Prism Big Dye Terminator v3.1 Cycle Sequencing Kit (Applied Biosystems) on an ABI Prism 3700 DNA Analyzer (Applied Biosystems). All sequences were analysed by the Chromas Software (Technelysium) [3].

Reforences

1 Liu, W.Y. *et al.*, Effect of aerobic exercise and low carbohydrate diet on pre-diabetic non-alcoholic fatty liver disease in postmenopausal women and middle aged men - the role of gut microbiota composition: study protocol for the AELC randomized controlled trial. *BMC Public Health* 14 (1), 48 (2014).

2 Hillsdale, N. & Erlbaum;, *Statistical power analysis for the behavioral sciences.* . (1988).

3 Luukkonen, P.K. *et al.*, Hepatic ceramides dissociate steatosis and insulin resistance in patients with non-alcoholic fatty liver disease. *Journal of hepatology* 64 (5), 1167-1175 (2016).
