# Supplementary material for: Effect of aerobic exercise and diet on liver fat in pre-diabetic patients with non-alcoholic-fatty-liver-disease: A randomized controlled trial
Source: Sci Rep. 2017 Nov 21;7:15952. doi: 10.1038/s41598-017-16159-x (PMC5698376; doi:10.1038/s41598-017-16159-x)
Supplement: Supplementary file 2 — Supplement Table S1 [file 41598_2017_16159_MOESM2_ESM.doc]

**Table S1. Mean (95% confidence interval) values of physical fitness and dietary intakes during intervention over 8.6-month period (ITT)**

| Variables | NI | | | | | AEx | | | | Diet | | | | | AED | | | | | Group by time | | | | |  | | |
| --- | --- | --- | --- | --- | --- | --- | --- | --- | --- | --- | --- | --- | --- | --- | --- | --- | --- | --- | --- | --- | --- | --- | --- | --- | --- | --- | --- |
|  | Baseline | | Intervention | | | Baseline | | Intervention | | Baseline | | | Intervention | | Baseline | | Intervention | | | Within G NI AEx Diet AED | | Between G 1-2 1-3 1-4 2-3 2-4 3-4 | | |  | | |
| VO2_max_ (ml/min/kg) | | 16.0 (14-18) | | 17.6 (16-20) | | | 15.4 (14-17) | | 19.2 (17-21) | | 17.8 (16-20) | | | 19.7 (18-22) | | 16.2 (14-18) | | 21.4 (19-23) | | | .102 <.001 .037 <.001 | | .103 .821 .012 .143 .311 .017 | | | | |
| Whole day | | | | | | | | | | | | | | | | | | | | | | | | | | | |
| Energy (Kcal/day) | | 1703(1553-1854) | | 1638(1495-1891) | | | 1767(1622-1911) | | 1627(1471-1784) | | 1769(1624-1913) | | | 1670(1513-1826) | | 1786(1648-1925) | | 1670(1481-1794) | | | .933 .188 .349 .153 | | .427 .585 .391 .785 .949 .735 | | | |  |
| Fiber (g/d)* | | 8.76 (7-11) | | 10.6 (8-13) | | | 8.90 (7-11) | | 10.2 (8-12) | | 12.2 (10-14) | | | 19.8 (18-22) | | 10.6 (9-13) | | 18.8 (17-21) | | | ..259 .327 <.001 <.001 | | .809 .006 .002 .001 <.001 .750 | | | |  |
| Protein (E%) | | 22.1 (20-24) | | 20.3 (17-23) | | | 19.3 (17-21) | | 22.4 (20-25) | | 20.3 (19-22) | | | 22.1 (20-24) | | 20.5 (19-22) | | 20.4 (18-23) | | | .309 .030 .193 .934 | | .030 .108 .461 .502 .102 .323 | | | |  |
| Carbohydrate (E%) | | 47.8 (43-53) | | 51.7 (46-58) | | | 50.8 (46-55) | | 51.7 (47-58) | | 51.2 (48-56) | | | 48.3 (43-53) | | 51.9 (48-56) | | 50.1 (45-55) | | | .296 .789 .346 .566 | | .535 .161 .242 .397 .556 .786 | | | |  |
| Fat (E%) | | 32.3 (28-37) | | 28.7 (24-34) | | | 30.7 (27-35) | | 27.3 (23-32) | | 29-2 (25-33) | | | 30.6 (26-35) | | 28.4 (25-32) | | 30.2 (26-34) | | | .283 .239 .601 .506 | | .976 .249 .212 .227 .191 .925 |  | | | |
| Lunch | | | | | | | | | | | | | | | | | | | | | | | | | | | |
| Energy (Kcal/day) | | 533 (470-596) | | | 505 (424-584) | | 483 (414-551) | | 533 (470-596) | | | 452 (384-521) | | 424 (369-479) | | 481 (414-547) | | | 462 (412-512) | | .734 .273 .512 .650 | | .331 .881 .994 .213 .263 .869 | | | |  |
| Fiber (g/d)* | | 2.72 (1.9-3.5) | | | 2.80 (1.9-3.7) | | 2.60 (1.9-3.3) | | 2.74 (2.1-3.4) | | | 3.49 (2.7-4.3) | | 11.3 (11-12) | | 3.02 (2.3-3.8) | | | 11.7 (11-12) | | .897 .765 <.001 <.001 | | .932 <.001 <.001 <.001 <.001 .155 | | | |  |
| Protein (E%) | | 20.2 (17-23) | | | 17.2 (15-19) | | 18.0 (15-21) | | 22.4 (21-24) | | | 21.4 (19-24) | | 26.3 (25-28) | | 21.9 (19-24) | | 26.5 (25-28) | | | .102 .004 .002 .002 | | .002 .001 .001 .830 .939 .884 | | | |  |
| Carbohydrate (E%) | | 50.4 (44-57) | | | 52.6 (47-58) | | 55.0 (49-61) | | 50.2 (46-54) | | | 53.0 (47-59) | | 38.3 (35-42) | | 51.4 (46-57) | | 38.0 (35-41) | | | .596 .165 <.001 <.001 | | .195 .002 .003 .036 .060 .782 | | |  | |
| Fat (E%) | | 32.5 (27-38) | | | 32.2 (28-37) | | 29.6 (25-35) | | 27.9 (25-31) | | | 26.2 (21-31) | | 36.5 (34-39) | | 27.5 (23-32) | | 36.9 (34-39) | | | .916 .577 <.001 .001 | | .768 .015 .023 .004 .006 .810 | | |  | |

Mixed model for repeated measures (2 factor interactions: group x time) followed by Sidak correction for within and between groups.

ITT=Intention to treatment analysis; NI = No intervention group; AEx=Exercise group; AED=AEx+diet group; E = energy.

*The dietary results are calculated on the basis of the China food composition, Book 1, 2^nd^ edition, National Institute of Nutrition and Food Safety, China CDC, edited by Yang Yuexin, Wang Guangya, Pan Xingchang, Peking University Medical Press, ISBN 978-7-81116-727-6, 2011. In this book, the calculation of fibre is only included insoluble fibre. The results of fibre for the diet and AED groups after intervention included also added 5 g of soluble fibre.
